# Low Salt Treatment Results in Plant Growth Enhancement in Tomato Seedlings

**DOI:** 10.3390/plants11060807

**Published:** 2022-03-18

**Authors:** Paola Rivera, Cristian Moya, José A. O’Brien

**Affiliations:** 1 Departamento de Genética Molecular y Microbiología, Facultad de Ciencias Biológicas, Pontificia Universidad Católica de Chile, Santiago 8331150, Chile; pbrivera@uc.cl (P.R.); camoya5@uc.cl (C.M.); 2 Departamento de Fruticultura y Enología, Facultad de Agronomía e Ingeniería Forestal, Pontificia Universidad Católica de Chile, Santiago 7820436, Chile

**Keywords:** *Solanum lycopersicum*, root growth, salt stress, plant growth

## Abstract

Climate change together with excessive fertilization and poor water quality can affect soil quality and salinization. In plants, high salinity causes osmotic stress, ionic toxicity, and oxidative stress. Consequently, salt stress limits plant development, growth, productivity, and yield. Tomatoes are a very common agricultural product, and some cultivars can partially tolerate salinity. However, most studies are focused on salt excess, which does not necessarily extrapolate on how plants develop in soils with low concentrations of salts. Thus, this study characterizes plant growth and the development of different salt concentrations from 25 to 200 mM in *Solanum lycopersicum* cv. Moneymaker. Tomato seedlings grown in Murashige and Skoog medium supplied with different NaCl concentrations (0, 25, 50, 75, 100, 125, 150, 175, and 200 mM) showed that low salt concentrations (25 and 50 mM) have a positive impact on lateral root development. This was further observed in physiological parameters such as shoot length, primary root length, and proliferation of lateral roots versus controls. Interestingly, no significant changes in Na^+^ concentration were observed in 25 mM NaCl in roots or shoots versus controls. Overall, our results suggest that non-toxic salt concentrations can have a positive impact on plant development.

## 1. Introduction

The continuous expansion of the global population demands a future increase in food production to maintain present caloric intake. Thus, researchers and growers need to adapt to climate change scenarios that threaten sustainability and food security. One of the main challenges is to overcome abiotic stresses. Under the current scenario, the yield of main crops can decrease by more than 50% worldwide [1]. Thus, biotechnology and modern breeding are promising alternatives to increase abiotic stress tolerance on crops. However, it is necessary to have a deeper understanding of the regulatory networks, tolerance mechanisms, and susceptibility of crops to these specific factors.

Salinity is a key problem in arid and semiarid regions [2]. Overall, it generates an osmotic and ionic stress that limits water intake and affects metabolic processes [3]. It also has an impact on the availability, transport, and distribution of various nutrients. Among the different salts, NaCl competes with other ions and is toxic for several species [4]. In tomatoes (*Solanum lycopersicum* L.), high salinity reduces protein content, carotenoids, chlorophyll, soluble solids, starch content, and phenolic compounds [5,6,7]. To counteract these detrimental effects, plants have a series of stress response mechanisms that are genetically encoded and involve ion exclusion, compartmentalization, and tissue prioritization [8,9,10]. 

High salt concentration in the soil can severely affect plant growth and yield due to the strong osmotic and ionic stress imposed on the root system. The osmotic stress caused by salt reduces cell expansion in growing tissues and also causes stomatal closure, which helps to minimize water loss and plant damage [11]. However, ions accumulating in plants can affect water availability and produce high toxicity and developmental constraints [11]. 

Although the above-ground tissues are affected under salt stress, roots are the main responsive organ to stresses such as high salt, and its growth and development are also altered. Roots are also the first tissue to sense changes in soil conditions [12]. Accordingly, the result of salt stress in roots is a severe re-adjustment of its morphology, which involves complex hormonal crosstalk [13,14,15,16]. The high plasticity of the root is controlled post-embryonically by changing the length of the primary root and the number and density of lateral roots (LRs), thus leading to a constant reduction in root growth in response to salt. Interestingly, *Arabidopsis thaliana* has contrasting effects in root development depending on the salt (NaCl) concentration applied to the roots [11,17]. While the root length shortens with increasing NaCl concentration, the number of LR increases at mild salt stress (<50 mM NaCl) and decreases drastically at severe stress (>100 mM NaCl) in Col-0 (wild type) [11,17]. 

To date, there are only a limited number of reports showing positive impacts of root traits under salt stress in tomatoes. A comparative study of cultivated and wild tomato species showed the variability of the root phenotype in response to salt stress [18]. While cv Rutgers increases its root length at 100 mM NaCl, cv Moneymaker showed only a minor change, and cv aichi-first is completely sensitive. A priming treatment of tomatoes (cv momotaro haruka) with 300 mM NaCl for 24 h before germination can have a positive impact on seed germination and root length [19]. Low salt stress applied in a non-uniform manner to the root system results in enhanced leaf growth and fruit yield [20]. 

Here, we establish an in vitro system for tomato seedlings to evaluate the plant response to a range of NaCl concentrations. We evaluated root and shoot growth parameters and identified contrasting salt concentrations, demonstrating a positive impact of low salt treatments on seedling growth.

## 2. Results

**Figure 1:**
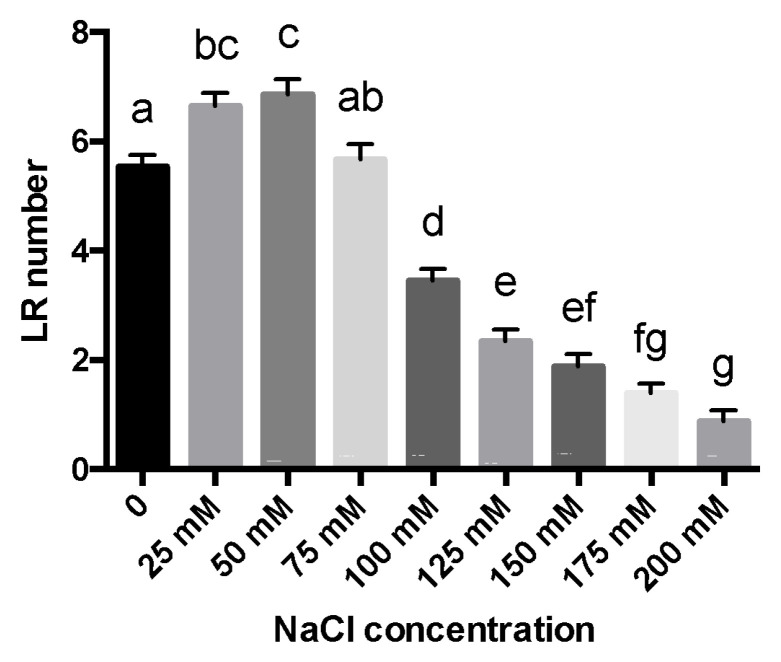
Number of lateral roots in response to salt stress. *S. lycopersicum* seedlings were treated with increasing concentrations of NaCl (0, 25, 50, 75, 100, 125, 150, 175, and 200 mM). The lateral root number (LR number) was measured after 10 days of NaCl treatment. The letters a–g represent statistically significant differences with *p* < 0.05. Error bars represent the standard error of the mean.

To evaluate the concentration-dependent effect of salinity on root development, we first focused on lateral root (LR) number under a range of NaCl concentrations (25–200 mM). Figure 1 shows that concentrations of 100 mM NaCl and above have a negative impact on the number of lateral roots. Interestingly, plants grown under NaCl at a concentration as low as 25 mM NaCl had significantly more lateral roots than controls (Figure 1). The same was observed for 50 mM NaCl. 

**Figure 2:**
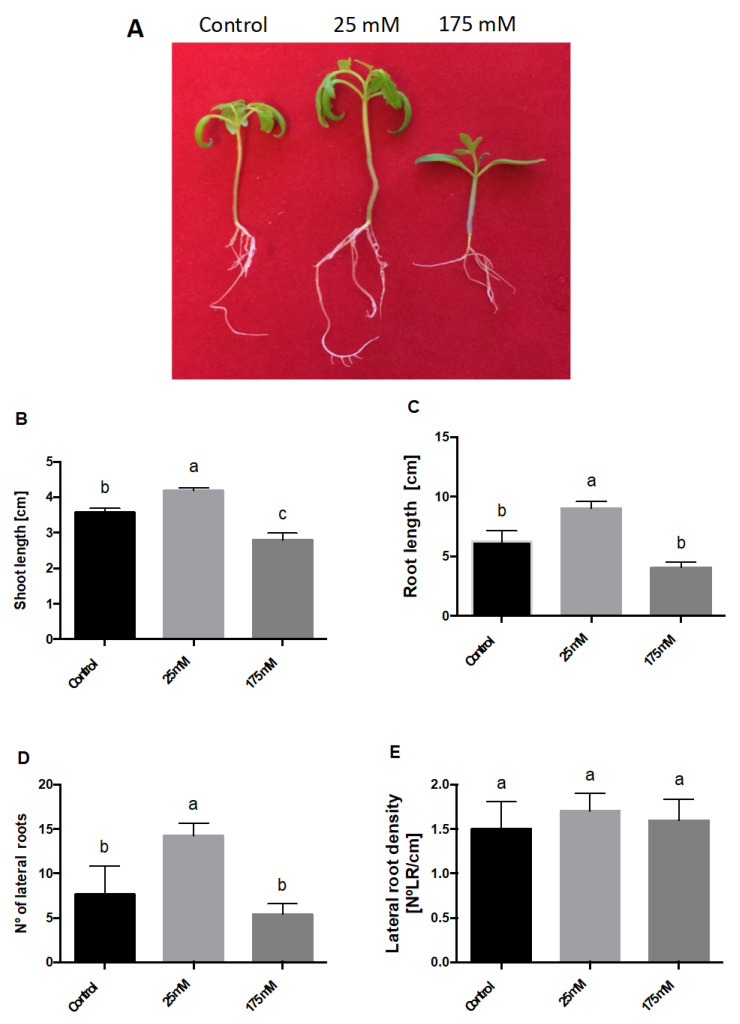
Low and high salt treatments show contrasting phenotypes in plant development. *S. lycopersicum* seedlings were treated with low and high concentrations of NaCl (0, 25, and 175 mM) for 10 days. (**A**) Representative picture of the seedlings after treatment, (**B**) shoot length, (**C**) root length, (**D**) number of lateral roots, and (**E**) lateral root density. The letters a–c represent statistically significant differences with *p* < 0.01. Error bars represent the standard error of the mean.

Figure 2 shows that low NaCl treatment had a positive impact on not only the number of LRs, but also other physiological parameters (Figure 2A). To characterize this further, we used two contrasting NaCl concentrations. The lower and higher NaCl concentrations leading to a significant phenotype served as the low (25 mM NaCl) and high (175 mM NaCl) salt concentrations, respectively. Interestingly, while 175 mM NaCl treatment resulted in a negative impact on shoot and root length, low salt had a positive impact, showing significantly longer shoots and roots (Figure 2B,C). There were more lateral roots with lower salt levels (Figure 2D). However, there were no variations with respect to the lateral root density; thus, the number of lateral roots per cm of root length remained constant (Figure 2E). 

**Figure 3:**
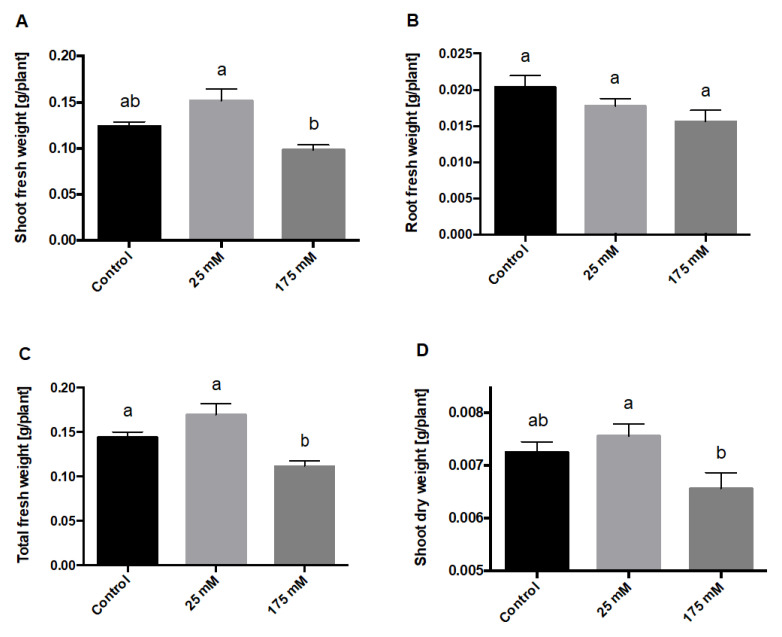
Fresh and dry weight of *S. lycopersicum* plants after low and high salt treatments. *S. lycopersicum* seedlings were treated with low and high concentrations of NaCl (0, 25, and 175 mM). The roots and shoots were dissected and weighed after 10 days of NaCl treatment. (**A**) Shoot fresh weight, (**B**) root fresh weight, (**C**) total fresh weight, and (**D**) shoot dry weight. The letters a and b represent statistically significant differences with *p* < 0.05. Error bars represent the standard error of the mean.

Considering the remarkable phenotype observed here, we evaluated higher seedling growth under low salt, as reflected in fresh and dry weights. Interestingly, while no changes were observed in root fresh or dry weight (Figure 3B and Figure S1), there was a clear trend (although not statistically significant) in the shoot and total plant fresh weight at 25 mM and 175 mM NaCl versus control. Moreover, there was a significant difference in shoot and total plant fresh weight between high and low salt concentrations (Figure 3A,C). We further evaluated these differences in terms of shoot dry weight, observing similar results (Figure 3D). 

**Figure 4:**
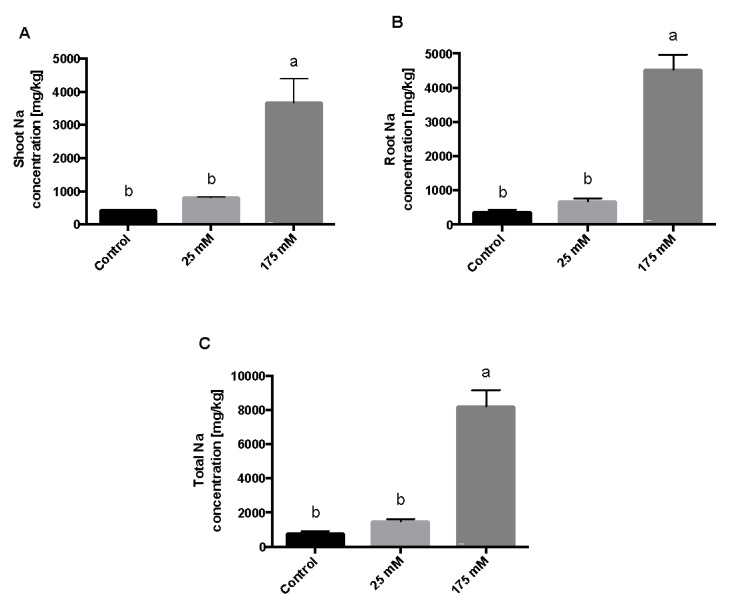
Na concentration in *S. lycopersicum* plants after low and high salt treatments. *S. lycopersicum* seedlings were treated with low and high concentrations of NaCl as well as controls (0, 25, and 175 mM). After 10 days of NaCl treatment, Na concentration was measured in the shoot (**A**), root (**B**), and total Na concentration (**C**). The letters a and b represent statistically significant differences with *p* < 0.05. Error bars represent the standard error of the mean.

Finally, the Na^+^ ion content in the root, shoot, and total Na^+^ concentration only changed under the 175 mM NaCl treatment (Figure 4). Thus, we found no significant differences in Na^+^ concentrations versus control at 25 mM NaCl. 

## 3. Discussion

Salt stress negatively impacts plant growth and development in a concentration-dependent manner. However, some reports suggest that lower salt concentrations might result in a positive effect [11,17,20]. Here, we observed a positive impact on lateral root number at 25 and 50 mM NaCl. There were no significant changes at 75 mM NaCl, and a negative impact at 100 mM and higher. These results are consistent with those described by Zolla et al. [17] in *Arabidopsis thaliana*, where they reported that adequate concentrations (non-toxic) of salt cause an increase in the number of lateral roots. They further argued that the stimulation of the number of lateral roots is due to the progression of an increased number of lateral root primordia from the pre-emergence stage to the lateral root formation stage. However, this might not be the case in tomatoes. Zolla et al. [17] reported a negative impact on root length, but we observed a positive impact. Thus, the higher number of lateral roots might result from an increase in the growth rate at the plant level. This is further supported by the significant differences observed between control and 25 mM NaCl in three of the four characteristics studied: length of the shoot (Figure 2B), length of the main root (Figure 2C), and number of secondary roots (Figure 2D). The lateral root density (Figure 2E) showed no significant difference between the control and NaCl treatments. This is because of the correlation of root length and the number of lateral roots, which increases or decreases proportionally.

Some researchers have suggested that moderate salinity can stimulate growth in some species [21], and it has been suggested that most crops are salt-sensitive during emergence and vegetative development [22]. In tomatoes, Srinieng et al. [23] reported a decreased growth of plants (cv. Puanghaka) at 5 mM NaCl. Interestingly, the length of the main root remained relatively constant even though the fresh weight of the roots decreased with increasing salt concentration. This is consistent with the results observed in this work, where the root fresh weight showed a decrease with increasing NaCl concentrations. These contradictory results are probably due to genetic variation among different cultivars and crop species [9].

Most studies do not test a wide range of concentrations, thus perhaps missing this positive effect. For example, de la Torre-González et al. [24] compared the response of two tomato genotypes under salinity stress: cultivar Grand Brix and cultivar Marmande Raf. The authors found that salt stress decreases the biomass and the relative growth rate in both cultivars. However, this effect is significantly greater in the cultivar Marmande Raf, thus reinforcing the hypothesis that mild salt responses are genetically dependent.

The positive impact of mild NaCl treatment in plant growth was supported by Na^+^ concentration in the plant. Our results show that only the plants exposed to toxic concentrations of NaCl (175 mM) accumulate significantly higher Na^+^. The concentration of Na^+^ ions was higher in the aerial part of the plant than in the root part for the three treatments, although the statistical analysis did not show a significant difference between them (*p* < 0.05). High concentrations of Na^+^ implies relevant stress for the plant because it affects gas exchange, chlorophyll fluorescence, and the availability of Ca^2+^ and K^+^ due to reduced transport and mobility [5]. These results agree with de la Torre-González et al. [24], who concluded that the decrease in biomass and the relative growth rate under salt stress is related to the accumulation of Na^+^ ions and the K^+^ deficit. On the other hand, Khaliq et al. [25] showed that salt stress affects several crops, such as alfalfa, due to the large accumulation of Na^+^ [25]. Cultivars or genotypes more tolerant to saline stress tend to accumulate less Na^+^; this is probably because these plants use strategies such as the compartmentalization of Na^+^ in the vacuoles or the immobilization of the ion [26].

## 4. Materials and Methods

### 4.1. Plant Material and Treatments

Tomato plants (*S. lycopersicum* cultivar Moneymaker) were germinated in vitro in half-strength MS medium [27] supplemented with 0.5 g L^−1^ MES, 10 g L^−1^ sucrose, and 0.8% agar (pH 5.9). Prior to germination, seeds were sterilized with a solution containing 0.1% triton and 2.5% sodium hypochlorite. Seeds were incubated under agitation for 10 min, followed by three washes with sterile distilled water. The seeds were later placed in Petri dishes for germination. Seeds were grown for 10 days in a growth chamber under controlled conditions (25 °C, 16 h photoperiod). Five days after germination, the seedlings were transferred to glass flasks (6 cm in diameter and 12 cm in height) containing solid MS medium (as described above). These were supplemented with increasing NaCl concentrations (0, 25, 50, 75, 100, 125, 150, 175, and 200 mM). The plants were removed for physiological characterizations and analysis after 10 days of NaCl treatment. The physiological characterization measured the shoot length, root length, number of lateral roots, and lateral root density (N° of lateral roots per cm of root length). The analysis measured the fresh weight, dry weight, and Na^+^ concentration.

### 4.2. Ion Concentration

Tomato seedlings were collected after 10 days of NaCl treatment. Root and shoot/leaf materials were collected separately. The roots were separated from the aerial part, and each material was oven dried for 48 h at 65 °C and weighed using an analytical balance (Precisa, Texas City, TX, USA). Cation concentrations were determined by dry combustion at 500 °C until the organic components turned to ashes. Ash tissue samples were dissolved in 2 M HCl, and concentrations were determined with an atomic absorption spectrophotometer (Varian SpectraAA 220 FS, Varian Techtron Pty. Limited; Mulgrave, VIC, Australia).

### 4.3. Statistical Analysis

All experiments were performed with at least three biological replicates. Each replicate consisted of at least five seedlings. Significant differences between treatments were analyzed using one-way ANOVA with a *p* < 0.05.

## 5. Conclusions

In conclusion, we established an in vitro system to evaluate various plant physiological parameters in response to low and high salt concentrations. The results suggest that the mechanism of salt stress response might vary depending on the salt concentrations. This system will be very helpful for further study of signaling mechanisms (particularly phytohormones) along with other parameters such as photosynthetic traits, redox homeostasis, and cell cycle regulation.

## Data Availability

Not applicable.

## Supplementary Materials

The following supporting information can be downloaded at: https://www.mdpi.com/article/10.3390/plants11060807/s1. Figure S1: Root dry weight of *S. lycopersicum* plants after low and high salt treatments. *S. lycopersicum* seedlings were treated with low and high concentrations of NaCl (0, 25, and 175 mM). After 10 days of NaCl treatment, roots were dissected, dried, and the root weight was calculated as g/plant. The letter a represents statistically significant differences with *p* < 0.05. Error bars represent the standard error of the mean.

## References

[1] Fahad S., Hussain S., Matloob A., Khan F.A., Khaliq A., Saud S., Hassan S., Shan D., Khan F., Ullah N. (2015). Phytohormones and plant responses to salinity stress: A review. Plant Growth Regul..

[2] Shahid S.A., Zaman M., Heng L. (2018). Soil salinity: Historical perspectives and a world overview of the problem. Guideline for Salinity Assessment, Mitigation and Adaptation Using Nuclear and Related Techniques.

[3] Tomescu D., Şumǎlan R., Copolovici L., Copolovici D. (2017). The influence of soil salinity on volatile organic compounds emission and photosynthetic parameters of *Solanum lycopersicum* L. varieties. Open Life Sci..

[4] Hu Y., Schmidhalter U. (2005). Drought and salinity: A comparison of their effects on mineral nutrition of plants. J. Plant Nutr. Soil Sci..

[5] Bonomelli C., Celis V., Lombardi G., Mártiz J. (2018). Salt Stress Effects on Avocado (*Persea americana* Mill.) Plants with and without seaweed extract (*Ascophyllum nodosum*) application. Agronomy.

[6] Leiva-Ampuero A., Agurto M., Matus J.T., Hoppe G., Huidobro C., Inostroza-Blancheteau C., Reyes-Díaz M., Stange C., Canessa P., Vega A. (2020). Salinity impairs photosynthetic capacity and enhances carotenoid-related gene expression and biosynthesis in tomato (*Solanum lycopersicum* L. cv. Micro-Tom). Peer J..

[7] Martínez J.P., Fuentes R., Farías K., Lizana C., Alfaro J.F., Fuentes L., Calabrese N., Bigot S., Quinet M., Lutts S. (2020). Effects of salt stress on fruit antioxidant capacity of wild (*Solanum chilense*) and domesticated (*Solanum lycopersicum* var. cerasiforme) tomatoes. Agronomy.

[8] Hendrik Poorte A., Nagel O. (2000). The role of biomass allocation in the growth response of plants to different levels of light, CO_2_, nutrients and water: A quantitative review. Aust. J. Plant Physiol..

[9] Ma T., Zeng W., Li Q., Yang X., Wu J., Huang J. (2017). Shoot and root biomass allocation of sunflower varying with soil salinity and nitrogen applications. Agron. J..

[10] Chaves M.M., Flexas J., Pinheiro C. (2009). Photosynthesis under drought and salt stress: Regulation mechanisms from whole plant to cell. Ann. Bot..

[11] Julkowska M.M., Hoefsloot H.C.J., Mol S., Feron R., De Boer G.J., Haring M.A., Testerink C. (2014). Capturing Arabidopsis root architecture dynamics with root-fit reveals diversity in responses to salinity. Plant Physiol..

[12] Jia W. (2002). Salt-stress-induced ABA accumulation is more sensitively triggered in roots than in shoots. J. Exp. Bot..

[13] Liu W., Li R.J., Han T.T., Cai W., Fu Z.W., Lu Y.T. (2015). Salt stress reduces root meristem size by nitric oxide-mediated modulation of auxin accumulation and signaling in Arabidopsis. Plant Physiol..

[14] Verma V., Ravindran P., Kumar P.P. (2016). Plant hormone-mediated regulation of stress responses. BMC Plant Biol..

[15] Vanstraelen M., Benková E. (2012). Hormonal interactions in the regulation of plant development. Annu. Rev. Cell Dev. Biol..

[16] Valenzuela C.E., Acevedo-Acevedo O., Miranda G.S., Vergara-Barros P., Holuigue L., Figueroa C.R., Figueroa P.M. (2016). Salt stress response triggers activation of the jasmonate signaling pathway leading to inhibition of cell elongation in Arabidopsis primary root. J. Exp. Bot..

[17] Zolla G., Heimer Y.M., Barak S. (2010). Mild salinity stimulates a stress-induced morphogenic response in *Arabidopsis thaliana* roots. J. Exp. Bot..

[18] Zaki H., Yokoi S. (2016). A comparative in vitro study of salt tolerance in cultivated tomato and related wild species. Plant Biotechnol..

[19] Nakaune M., Tsukazawa K., Uga H., Asamizu E., Imanishi S., Matsukura C., Ezura H. (2012). Low sodium chloride priming increases seedling vigor and stress tolerance to *Ralstonia solanacearum* in tomato. Plant Biotechnol..

[20] Wang W., Cai L., Long Z., Zhang X., Zhao F. (2021). Effects of non-uniform salt stress on growth, yield, and quality of tomato. Soil Sci. Plant Nutr..

[21] Bell H.L., O’Leary J.W. (2003). Effects of salinity on growth and cation accumulation of *Sporobolus virginicus* (Poaceae). Am. J. Bot..

[22] Läuchli A., Grattan S.R. (2007). Plant growth and development under salinity stress. Advances in Molecular Breeding toward Drought and Salt Tolerant Crops.

[23] Srinieng K., Saisavoey T., Karnchanatat A. (2015). Effect of salinity stress on antioxidative enzyme activities in tomato cultured in vitro. Pak. J. Bot..

[24] de la Torre-González A., Navarro-León E., Albacete A., Blasco B., Ruiz J.M. (2017). Study of phytohormone profile and oxidative metabolism as key process to identification of salinity response in tomato commercial genotypes. J. Plant Physiol..

[25] Khaliq A., Zia-ul-Haq M., Ali F., Aslam F., Matloob A., Navab A., Hussain S. (2015). Salinity tolerance in wheat cultivars is related to enhanced activities of enzymatic antioxidants and reduced lipid peroxidation. CLEAN—Soil Air Water.

[26] Li R., Shi F., Fukuda K., Yang Y. (2011). Effects of salt and alkali stresses on germination, growth, photosynthesis and ion accumulation in alfalfa (*Medicago sativa* L.). Soil Sci. Plant Nutr..

[27] Murashige T., Skoog F. (1962). A Revised medium for rapid growth and bio assays with tobacco tissue cultures. Physiol. Plant..

