# Supplementary material for: Low Salt Treatment Results in Plant Growth Enhancement in Tomato Seedlings
Source: Plants (Basel). 2022 Mar 18;11(6):807. doi: 10.3390/plants11060807 (PMC8954722; doi:10.3390/plants11060807)
Supplement: Supplementary file 1 [file plants-11-00807-s001.zip › plants-1618043-supplementary.pdf]

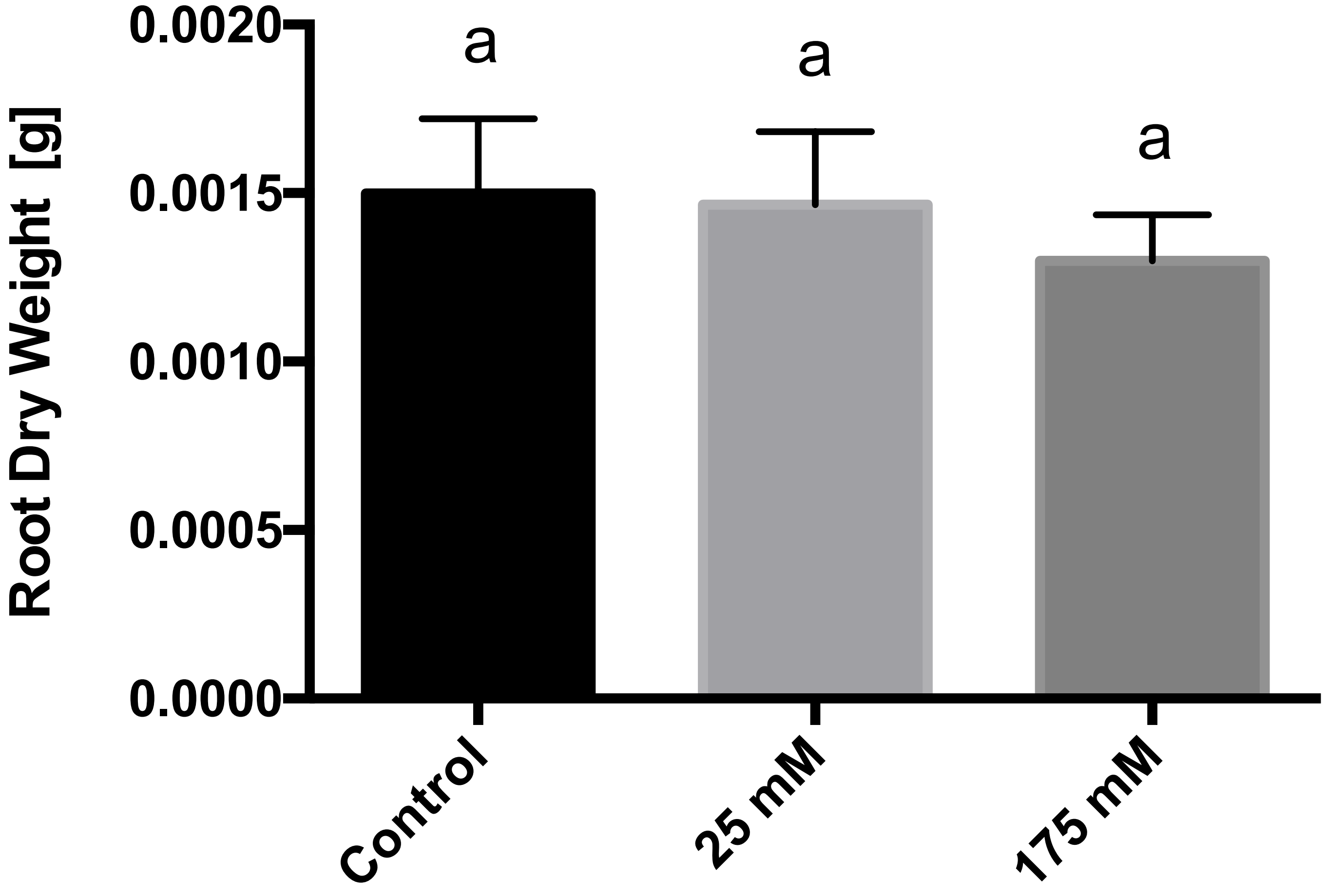


**Figure S1: Root dry weight of *S. lycopersicum* plants after low and high salt treatments.**

*S. lycopersicum* seedlings were treated with low and high concentrations of NaCl (0, 25, and 175 mM). After 10 days of NaCl treatment, roots were dissected, dried, and the root weight was calculated as g/plant. Here, "a" represents statistically significant differences with $p < 0.05$. Error bars represent the standard error of the mean.
